# Long-Term Effects of Environmental Lead Exposure on Blood Pressure and Plasma Soluble Cell Adhesion Molecules in Young Adults: A Follow-Up Study of a Prospective Cohort in Kosovo

**DOI:** 10.1155/2018/3180487

**Published:** 2018-01-08

**Authors:** Pashko R. Camaj, Joseph H. Graziano, Emine Preteni, Dusan Popovac, Nancy LoIacono, Olgica Balac, Pam Factor-Litvak

**Affiliations:** ^1^Department of Environmental Health Sciences, Mailman School of Public Health, Columbia University, New York City, NY, USA; ^2^Medical Center, Mitrovica, Kosovo; ^3^Medical Faculty, University of Prishtina, Prishtina, Kosovo; ^4^Department of Epidemiology, Mailman School of Public Health, Columbia University, New York City, NY, USA

## Abstract

**Background and Aims:**

Epidemiologic studies examining the relationship between environmental lead (Pb) exposure and blood pressure (BP) generally report small associations between blood lead concentration (BPb) and BP. However, these studies are predominantly cross-sectional. In addition, no epidemiologic studies evaluate associations between either current or past Pb exposure and serum levels of markers of systemic inflammation and endothelial dysfunction, including soluble vascular adhesion molecule (sVCAM-1) and soluble intercellular cell adhesion molecule (sICAM-1).

**We prospectively investigate these associations later in life:**

* Methods*. From our original prospective birth cohort study in Mitrovica (a mining town) and Prishtina (a control town), Kosovo, from 1985 to 1998, we located and assessed BPb and BP in 101 participants (mean age of 24.9 years old) in 2011.

**Results:**

We found highly statistically significant association between concurrent BPb and sVCAM-1 in men and a marginally significant association between concurrent PBb and sICAM.-1 in women. We did not find evidence of mediation.

**Conclusion:**

Current study results, along with previously reported findings on this cohort, provide evidence for the hypothesis that exposure to Pb leads to small increases in sBP and perhaps to increased circulating levels of sVCAM-1 and sICAM-1 later in life.

## 1. Introduction

Public health initiatives have been successful in dramatically reducing exposure to environmental lead (Pb), especially in the United States [1, 2]. However, even at low-levels of Pb exposure, there is support for an association between Pb and cardiovascular health and all-cause mortality among US population [3, 4]. A significant body of research has reported associations between blood Pb concentrations (BPb) and blood pressure (BP) in populations with BPb at levels that had until recently been considered as “safe” for adults (i.e., <10 *μ*g/dl) [5–8]. Support for a relationship between Pb and BP comes from a wide range of animal studies as well as clinical and epidemiological studies of Pb-exposed workers and the general population. A significant number of reviews and meta-analyses based on more than 30 studies and more than 50,000 participants have reached the prevailing conclusion that there is significant association between BPb and BP [9–16].

In addition, a limited number of published studies examining associations between Pb exposure and BP in children have reported contrasting results. In one cross-sectional study, no association was observed between BPb and BP in children [17]. Increasing* cord* BPb level was associated with significantly higher baseline sBP and marginally higher baseline dBP at 9.5 years of age [18]. Maternal bone Pb (tibia-Pb) was associated with increase in sBP and dBP only in girls [19]. Analyses of the BPb and BP relationship at age 5.5 in the Yugoslavia study of lead exposure, pregnancy outcomes, and child development found small, non-statistically significant associations between BPb and both sBP and dBP [20]. Although modest, there is some evidence that prenatal Pb exposure may be associated with BP later in life. Here, we extend these findings by evaluating the associations between prenatal and childhood lead exposure and BP in early adulthood.

We also examine associations between BPb and two markers of inflammation and endothelial dysfunction, sICAM-1 and sVAM-1, both of which are proposed as mechanisms for the Pb-BP associations. Both markers are associated with CVD risk along with factors such as hypertension, smoking, and frequent alcohol consumption [21, 22] and are related to increasing sBP [23].

## 2. Methods

### 2.1. Study Design

The original cohort has been previously described [24, 25]. Briefly, pregnant women were recruited between 1984 and 1985 in two towns, Mitrovica, the site of the Trepça mines, smelter, and battery plant, and Prishtina, the capitol which was relatively unexposed. Offspring were followed every 6 to 12 months for BPb measures, neurocognitive development, and physical examinations until age 12.5. We located and identified 101 members of the original cohort from the Yugoslavia study of environmental lead, pregnancy outcomes, and child development and requested their participation in a follow-up study, in which each participant would be evaluated once to assess their BPb, BP, and serum sICAM-1 and sVCAM-1. Subjects were recruited through television, radio, and newspaper advertising and through “word of mouth.” Following a procedure to ensure that they were from the original cohort, we set up appointments for all participants to report to a central location in each town in order to conduct interviews, fill-out questionnaires, and collect biological samples. All questionnaires were administered in the participant's primary language, Albanian or Serbian. While we contacted and recruited study participants in both towns, we encountered more challenges finding and recruiting the participants from Prishtina. This may be due to the fact that the city expanded greatly in the past 8–10 years with changes in the ethnic makeup of the population. In addition, once contacted, those in Prishtina were less interested, and more of them refused to take part in the study, likely due to perception of the beneficial aspects of the study. Another factor that may have influenced the recruitment may have been that the overwhelming majority of the prewar Serbian population in Prishtina had been uprooted from Kosovo at the beginning of NATO campaign in 1999. However, the Serbian population in Mitrovica (Northern part of the city) for the most part has remained in place. There may have been some temporary migration during the few months at the height of the war, but it has been reported that most had returned to their homes shortly after. Similarly, the Albanian population in Mitrovica has remained very much intact with a similar pattern of temporary migration during the few months (1–3 months) of the height of the hostilities. Their temporary migration was mostly to camps in neighboring Albania and Montenegro. However, as the hostilities diminished, the overwhelming majority returned to their homes.

### 2.2. Data Collection and Laboratory Analysis

Demographic information and other lifestyle characteristics such as smoking were collected via questionnaire. We accessed existing data from the mid-pregnancy, delivery, and childhood questionnaires to ascertain the lifestyle characteristics of the mothers of cohort members as needed. Data on the outcomes were collected as follows:

- BP was measured using an automated monitor three times (at 1-minute intervals) at the completion of the interview and questionnaire but after the blood draw (Omron BP Monitor, Model # 785, Lake Forest, Illinois). For the statistical analysis, we used the standard research method [26] of taking three measurements and using the average of the two last measurements. As anticipated, the first BP measure was higher than subsequent measures; the mean differences between the first and third measures were 2.53 mmHg (*p* < 0.001) and 2.33 (*p* < 0.001) for sBP and dBP, respectively. This is likely due to the “white coat” effect [27, 28].
- Blood samples were collected in EDTA vacutainers by venipuncture from each participant. BPb levels were analyzed using a Graphite Furnace Atomic Absorption Spectrophotometer (GFAAS), model AAnalyst 600 (Perkin-Elmer, Shelton, CT) using a method modified after [29]. Columbia University's laboratory participates in the Center for Disease Control and Prevention quality control program for BPb; in the past three years, the interclass correlation between the expected and observed BPb was 0.99.
- Human sICAM-1 and sVCAM-1: Serum levels sICAM-1 and sVCAM-1 were analyzed utilizing the Quantikine Human sICAM-1/CD54 and sVCAM-1 kits that included precoated microplates, standard, calibrator diluent, wash buffer, color reagents, and “stop” solution (R&D Systems, Minneapolis, MN). Interprecision coefficients of variation for sVCAM-1 were 4.6% for quality control samples and 5.7% for study samples. The corresponding values for sICAM-1 were 2.8% and 2.5%.

### 2.3. Statistical Analysis

We first conducted descriptive analyses to compare the distributions of variables of interest between towns and assessed differences using *t*-test or chi-square tests, and unadjusted bivariate linear regression models relating exposure to outcomes as appropriate. Linear regression was used to model associations between BPb and BP, sICAM-1, and sVCAM-1 controlling for ethnicity, sex, body mass index (BMI), smoking history, employment status, and education. In secondary analyses, we stratified by sex. Potential confounders were retained in the final models if the estimated coefficient relating concurrent BPb to BP, sVCAM-1, and sICAM-1 changed at least 10% with their inclusion. In addition, we also assessed for evidence of mediation by sVCAM-1 and sICAM-1; a reduction in the estimated regression coefficient relating BPb to BP with sVCAM-1 and/or sICAM-1 in the model was regarded as evidence of mediation.

BPb measures were available for each subject from birth through 12.5 years of age, and at the follow-up. We first examined the associations with BPb measured at the time of follow-up, concurrent BPb. Second, we calculated cumulative lead exposure, using the trapezoidal area under the curve for various age periods. We calculated the area under the curve for exposure between ages 0 and 2, ages 2 and 4, ages 4 and 7, and ages 7 to 12. Third, we also examined the associations between BP and the total area under the BPb versus age curve (AUC). All analyses were performed using SAS version 9.3 (SAS Institute, Carey, NC).

## 3. Results

**Figure 1:**
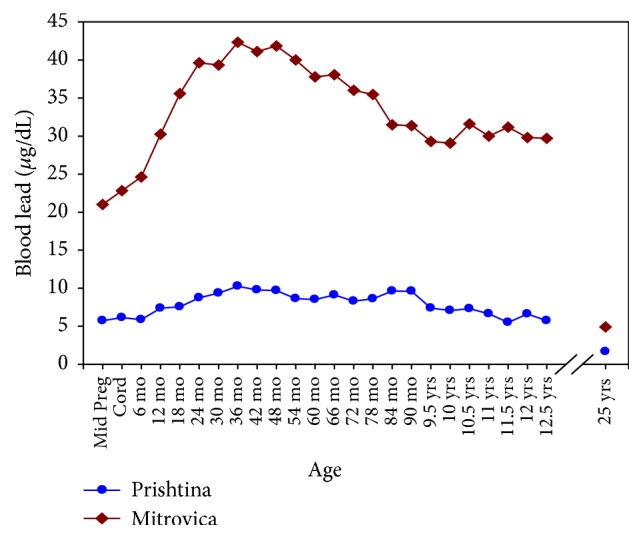
Average BPb levels in Mitrovica (top) and Prishtina (bottom) for the first 12.5 years of their lives and at 25 years of age (*N* = 101).

The original cohort consisted of 574 children; the sample size was reduced to 178 at the follow-up at age 12.5 years, likely because of the wartime situation. From the original 574 children cohort, we assembled 101 participants in 2011 when they were approximately 25 years old. Over 80% of the follow-up sample were from Mitrovica compared to 55% in the original birth cohort. Those residing in Prishtina were better educated and more likely to be employed. Smoking was more prevalent in Prishtina (66% versus 29% in Mitrovica). The sex distribution reflected that of the original cohort. Biomarkers and anthropometric characteristics are also listed in Table 1. Mean BPb concentrations remained significantly higher in Mitrovica residents than in those from Prishtina, even though the lead smelting plant drastically diminished its operations in the late 1990s and closed at the onset of the war in 1999. The smelting operations have remained closed and only ore mining operation at reduced capacities has resumed since 1999. Average BPb concentrations between birth and age 12.5 and at age 25 are illustrated in Figure 1. At age 12.5, the mean BPb concentrations were 30.6 *μ*g/dl (SD = 8.8 *μ*g/dl) in Mitrovica and 6.1 *μ*g/dl (SD = 1.6 *μ*g/dl) in Prishtina. Concurrent BPb levels ranged from 1.41 to 16.4 *μ*g/dl and 0.69 to 3.51 *μ*g/dl in Mitrovica and Prishtina, respectively. Mean sBP were 129.90 (SD = 14.87) mmHg and 125.64 (SD = 9.45) mmHg in Mitrovica and Prishtina, respectively (Table 2). Mean dBP were 81.31 (SD = 7.51) and 79.00 (SD = 5.09) in Mitrovica and Prishtina, respectively. Higher sBP and dBP were found among Serbians, males, those with higher body mass index (BMI), those with lower education, and nonsmokers.

**Figure 2:**
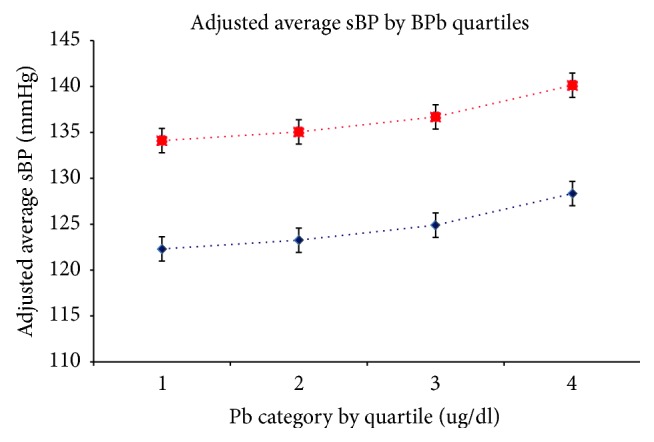
Linear relationship of concurrent BPb Quartiles and sBP for a smoker with high BMI (top) and a nonsmoker with low BMI (bottom).* Adjusted for smoking, BMI, sex, ethnicity, and education.*

Results from the regression models relating BPb to sBP and dBP are shown in Table 3. After adjusting for all potentially confounding variables, the magnitude of the regression coefficient diminished to 0.98 mmHg (95% CL 0.09, 1.86) increase in sBP per log unit increase in BPb. This relationship is illustrated in Figure 2. Furthermore, after stratifying by sex, adjusted regression coefficient relating BPb to sBP for males was 1.07 mmHg (95% CI −0.13, 2.26) and for females was 0.68 mmHg (95% CI −0.73, 2.10). The adjusted regression coefficient relating BPb to dBP was 0.35 mmHg (95% CI −0.11, 0.81) and it was for each log unit increase in BPb. After stratifying by sex, the adjusted regression coefficient relating BPb to dBP for males was 0.54 mmHg (95% CI −0.07, 1.14) and for females was 0.05 mmHg (95% CI −0.82, 0.72).

We also examined the associations between sBP and the total area under the BPb versus age curve (AUC) for postnatal periods of birth to ages 2, 2–4, 4–7, and 7–12.5 years. The associations between BPb and specific postnatal age periods were not significantly associated with either sBP or dBP, (shown in Table 4).

**Figure 3:**
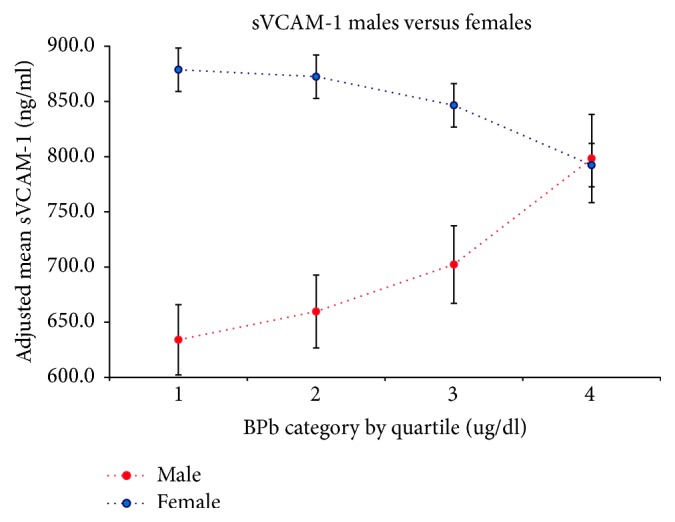
Linear relationship between concurrent BPb Quartiles and sVCAM-1 in males (bottom) and females (top).* Adjusted for smoking, BMI, ethnicity, and education.*

The unadjusted regression coefficient relating concurrent BPb to sVCAM-1 was 12.92 ng/ml (95% CI −1.32, 27.17) for each unit increase in BPb (Table 5). However, after stratifying by sex and adjusting for BMI, ethnicity, education, and smoking, the magnitude of the regression coefficient in men increased and was statistically significant 27.0 ng/ml (95% CL 9.74, 44.3) for each unit increase in BPb. The unadjusted regression coefficient for sICAM-1 was statistically significant at 4.76 ng/ml (95% CL 1.14, 8.38) for each log unit increase in BPb. After stratifying by sex and adjustments, the regression coefficient for men declined to 3.63 ng/ml (95% CL −0.74, 7.46) for each log unit increase in BPb. In women, the regression coefficient was marginally significant at 5.81 ng/ml (95% CL −0.05, 11.68). This relationship is illustrated in Figure 3. In addition, we examined the relationship between sBP and sVCAM-1 and the association was not significant (Supplemental Table (available here)).

Lastly, we also examined the associations between sVCAM-1 and sICAM-1 and the total area under the BPb versus age curve (AUC) for postnatal periods of birth to ages 2, 2–4, 4–7, and 7–12 years (data not shown). These measures of BPb were not associated with either sICAM-1 or sVCAM-1. We also examined the relationship between sBP and sVCAM-1 and the association was not significant (Supplemental Table).

## 4. Discussion

We tested the hypothesis that BPb measured in early life and at the same time as the outcome measure is associated with an increase in BP later in life. Past lead exposure was estimated using available past measurements of BPb from birth through age of 12. Between ages 12 and 25 years, the mean BPb fell from 29.7 to 4.91 *μ*g/dl in Mitrovica and from 5.73 to 1.67 *μ*g/dl in Prishtina, most probably due to the demise of the lead industry in the area. Increased concurrent BPb was associated with sBP for all participants, 0.98 mmHg (95% CL 0.09, 1.86) per log unit increase in BPb, which is similar to that in other studies [9, 10, 12–15, 30]. Furthermore, the association was marginally stronger in men compared to women, but neither was statistically significant, likely due to small sample size. The effects of BPb on BP have been shown to differ between men and women, albeit in different directions depending on the study. Some find larger associations between BPb and BP in women [7, 16, 30] and others in men [3, 11, 31, 32]. We also examined the associations between BP and the total area under the BPb versus age curve (AUC) as well as the AUC for early postnatal periods (birth to ages 2 years, 2–4 years, 4–7 years, and 7–12.5 years). While we found positive association between concurrent BPb and sBP and dBP, earlier measures of BPb were not significantly associated with either BP measure; however small observed associations suggest that sustained exposure may be more important for this outcome.

In addition, the association between BPb and circulating levels of two markers of endothelial cell dysfunction, sVCAM-1 and sICAM-1, suggested that BPb may be associated with inflammatory markers. We found a highly significant association between concurrent BPb and sVCAM-1 in men. To our knowledge, this is the first study that has evaluated this association. Although associations between sVCAM-1 and blood pressure are found in several studies these are cross-sectional and do not allow temporal inferences [22, 33, 34]. Our study did not find an association between sBP and sVCAM-1.

Accumulating scientific evidence in several populations suggests that exposure to Pb may result in increased BP. In its review of many epidemiological and occupational studies, the US Environmental Protection Agency concluded that every doubling of BPb levels is associated with a ~1.0 mmHg increase in sBP and ~0.6 mmHg increase in dBP [15], magnitudes close to those found in the present study. Early studies, including three large population-based studies [the British Regional Heart Study (BRHS) [35], the National Health and Nutrition Examination Survey (NHANES II and III) [6, 30–32], and Welsh Heart Programme study [36]], observed associations between BPb levels and increased BP. Several meta- and semiquantitative analyses reported similar relationships between BPb and BP [9–15]. In a meta-analysis of 23 studies, a doubling of BPb from 5 to 10 *μ*g/dl was associated with increases in sBP and dBP (1.0 mmHg and 0.6 mmHg, resp.) [12]. Another meta-analysis comprised 15 publications reported an average of 1.25 mmHg of sBP increase for every doubling of BPb concentration [11]. Our findings add to a number of earlier cross-sectional analyses that reported increases in sBP and dBP in relation to BPb across various populations [3, 31, 32, 35], including pregnant women [37].

An association between bone Pb and BP has also been reported. Bone Pb levels had a stronger association to BP and hypertension (defined as repeated measurements of >140/90 mmHg) than to BPb level in adult men [38, 39].

Studies examining associations between Pb exposure and BP in children have reported differing results. Among members of this study's cohort at 5.5 years of age a 10 *μ*g/dl increase in BPb was associated with a small increase of 0.5 (95% CL −0.2, 1.3) mmHg for sBP and a small increase of 0.4 (95%, CL −0.1, 0.9) mmHg dBP [20]. No association was observed between increased BPb levels and BP in a cohort of 149 children in Philadelphia (1–10 years old) [17]. Another study reported that increasing* cord* BPb levels were associated with significantly higher baseline sBP and marginally higher baseline dBP in children at 9.5 years of age, while no association between BP and* postnatal* BPb levels was found [18]. A clinical study of 780 children also found no association between BPb and BP [40]. More recently, Zhang [19] found that* maternal* bone Pb (tibia-Pb) was associated with increase in sBP and dBP only in girls and no associations were found between cord and* postnatal* (early childhood) BPb levels and BP.

The biological plausibility of the BPb and BP relationship has been documented in a number of animal studies. In rats, exposure to Pb for 4-5 months (leading to BPb concentration of 30–40 *μ*g/dL) was reported to induce hypertension [41, 42]. More recently, sBP increased in rats after exposure to 90–10,000 ppm Pb (as Pb-acetate in drinking water) for various time periods that resulted in BPb levels between 19.3 and 240 *μ*g/dL [43]. The disruption of the biological functions that can interfere with tightly regulated processes such as cell signaling, intracellular ion homeostasis, ion transport, energy metabolism, and enzyme functions is one of possible ways of Pb-induced cardiovascular toxicity. One plausible mechanism concerns the adverse effects of Pb on the kidney renin-angiotensin system [41]. In addition, Pb may affect sites of the cardiovascular system that control heart excitability and contractility or may impact compartments of the central nervous system that regulate BP and other cardiovascular functions [5, 44]. Pb-induced oxidative stress via generation of reactive oxygen species (ROS) has been thought to be a primary contributory factor in the pathogenesis of its adverse health effects [45]. A number of studies have demonstrated a role for oxidative stress in the pathogenesis of Pb-induced hypertension, mediated by the inactivation of nitric oxide (•NO) and down-regulation of soluble guanylate cyclase (sGC) [45, 46]. The reduction of the vasodilator •NO can lead to increased vasoconstriction and subsequently BP.

It has also been reported that Pb may be an important factor of stimulation and proliferation of vascular smooth muscle cells [47]. Recent studies indicate that the concentrations of the circulating sVCAM-1 and sICAM-1 appear to have predictive value for the identification of early atherosclerotic lesions and future cardiovascular disease (CVD) [48–52]. These adhesion molecules play a crucial role in the immune system response by promoting cell–cell and cell–stroma interactions and leukocyte migration [53]. The process of adhesion of the leukocytes to the endothelial cells and subsequent transendothelial migration is an important step in the atherosclerosis, arthritis, and cancers [54, 55]. While studies have shown that age is the most powerful independent predictor of the increasing levels of sICAM-1 and sVCAM-1, the effects of Pb may also play a significant role in this process [56]. Elevated sICAM-1 levels have been associated with CVD risk factors such as hypertension, smoking, and frequent alcohol consumption [21, 22] and were found to be associated with increased sBP. These associations, however, were found in cross-sectional data and it cannot be inferred that elevated BP results in inflammation, subsequently manifested by elevated levels of sICAM [23]. Studies in otherwise healthy men reported associations between increased levels of sICAM-1 and risk of future myocardial infarction [57, 58], suggesting the increased levels of sICAM-1 to be cellular mediators of inflammation. Angiotensin II, which is a potent vasoconstrictor, stimulates the sICAM-1 expression and, as such, it may play a role in the increase in BP [59]. Overall our study found a modest association between BPb levels sICAM-1 and sVCAM-1. However, the association was highly significant in men. There is limited information on this, but a few studies indicate that the female sex hormones may play a possible protective role on the vascular endothelial function, but the exact mechanisms of such protection are not known. In a study by Bonello and Norman (2002) [60] sICAM-1 levels were maximal in the early and mid-follicular stages and progressively decreased throughout the remainder of the cycle. sVCAM-1 levels also declined in the luteal phase. In addition, VCAM-1 shows better agreement regarding surface expression and liberation to the media than ICAM-1 did. The concentrations of sVCAM-1 are also higher than of sICAM-1, rendering changes easier to measure [61], which could explain the different outcome between these two biomarkers. This finding along with the reported slight increases in the sBP may be early sign indicating negative effects of Pb on cardiovascular system. However, we did not find sICAM-1 or sVCAM-1 to be a mediator in the relationship between BPb and sBP (data not shown).

This study has several strengths. First, there was a wide range of BPbs measured early in life, albeit less of a range at age 25, making possible the analysis of early life exposure and later outcomes. Second, the sample has few adult risk factors for high BP; that is, over 70% had BMIs between 20 and 24.9 and most were “light” smokers (smoking a few to <20 cigarettes a day). Third, although the follow-up was small, baseline characteristics of the participants in each town were similar to those from the larger pregnancy cohort. The study is limited in that we only followed a select 20% of the original sample; however, there is no reason to assume that the biological relationships would differ between those followed and those not followed. Another limitation of this study is the lack of data on usual diet. However, Kosovo is a small country with a relatively homogenous population. Diet was not included in the questionnaire because there are few dietary options. According to a 2002 UNICEF report, white bread is a staple food consumed almost every day in Kosovo [62]. Eggs, fruits, yoghurt, and dairy products were also frequently consumed with a frequency of consumption of five days a week. Meat and vegetables were consumed three times a week; beans or lentils were used in the meals twice a week while fish, spinach, and dark bread were consumed less than once a week. A third limitation pertains to the single measure of CVD risk markers. Fourth, there was a 12.5-year gap in the BPb measurements. Fifth, we had limited statistical power to find small associations. Finally, our analysis is based on a select population residing in Kosovo and the results may not be generalizable to other populations.

## 5. Conclusion

We found a statistically significant association between BPb measured concurrently and sBP, and a marginally significant association between BPb and dBP, which is consistent with previous studies. Furthermore, our data showed a significant association between concurrent BPb and levels of circulating sVCAM-1 in men, and a suggestive association between concurrent BPb and levels of circulating sICAM-1 in women, possibly a mechanism by which Pb may lead to increased BP. The findings support the hypothesis that the exposure to Pb poses a risk for elevated BP.

## Figures and Tables

**Table 1:** Sample characteristics [(%) or mean ± SD]. Follow-up of participants in the Yugoslavia Study of Lead Exposure and Child Development.

Variable	Prishtina (*N* = 21)	Mitrovica (*N* = 80)	*p* value
	*N* (%)	*N* (%)	
*Mean age*	24.9 + 0.49	24.8 + 0.48	0.49
*Ethnicity*			
% Albanian	95.2	68.7	0.01
% Serbian + other	4.8	31.2	
*Sex*			
% Male	47.6	46.2	0.91
% Female	52.4	53.7	
*Education*			
% High school or less	19.0	51.3	0.01
% College or more	80.9	48.7	
*Employment Status*			
% Employed	76.2	47.4	0.02
% Unemployed/searching	23.8	52.5	
*Smoking status*			
% Current	66.6	29.3	0.01
% No	33.3	70.7	
*Maternal Education*			
% High school or less	85.7	93.7	0.22
% College or more	14.3	6.2	
Mean BMI^*∗*^	23.0 ± 2.3	24.0 ± 4.5	0.15
Mean height (m)	1.7 ± 0.09	1.7 ± 0.09	0.55
Mean concurrent BPb^*∗∗*^ (*µ*g/dl)	1.7 ± 0.7	4.9 ± 3.26	<0.0001
Mean concurrent Hgb^*∗∗∗*^ (g/dl)	14.0 ± 1.7	13.6 ± 1.71	0.36
Mean concurrent sVCAM-1^#^ (ng/ml)	659.1 ± 170.6	734.4 ± 241.2	0.20
Mean concurrent sICAM-1^∧^ (ng/ml)	202.4 + 79.6	220.9 + 53.7	0.35

^*∗*^Body mass index. ^*∗∗*^Blood lead. ^*∗∗∗*^Hemoglobin. ^#^Soluble vascular adhesion molecules. ^∧^Soluble intercellular adhesion molecules.

**Table 2:** Demographic and other selected characteristics by blood pressure and blood lead levels follow-up of participants in the Yugoslavia Study of Lead Exposure and Child Development.

	Systolic Blood pressure		Diastolic Blood pressure		Blood Lead levels	
	Mean SD	*p* value	Mean SD	*p* value	Mean SD	*p* value
*Ethnicity*						
Albanian (*N* = 75) Serbian and 5 “other” (*N* = 26)	128.5 (9.8) 130.3 (22.1)	0.56	80.5 (5.7) 81.7 (10.1)	0.43	3.7 (2.8) 5.8 (3.6)	0.0028
*Sex*						
Male (*N* = 47) Female (*N* = 54)	131.9 (14.8) 126.3 (12.7)	0.04	82.3 (7.5) 79.5 (6.5)	0.04	4.6 (3.6) 3.9 (2.7)	0.33
*Education*						
High school or less (*N* = 45) College (*N* = 56)	131.6 (17.6) 126.8 (9.7)	0.08	81.3 (8.4) 80.4 (5.8)	0.56	5.4 (3.6) 3.3 (2.4)	0.0006
*Smoking*						
Current (*N* = 36) No (*N* = 60)	125.8 (11.2) 131.2 (15.4)	0.071	79.4 (6.3) 81.5 (7.1)	0.13	3.6 (3.1) 4.4 (3.2)	0.23
*BMI*						
20–24.9 (*N* = 65) 25–29.9 (*N* = 36)	123.9 (9.7) 138.7 (15.9)	0.0001	78.4 (5.3) 85.3 (7.8)	0.0001	3.6 (2.7) 5.5 (3.8)	0.009
*Location*						
Mitrovica (*N* = 81) Prishtina (*N* = 21)	129.9 (14.8) 125.4 (9.4)	0.20	81.3 (7.5) 79.0 (5.05)	0.18	4.9 (3.2) 1.7 (0.6)	0.0001

**Table 3:** Linear regression models for concurrent bpb on systolic and diastolic blood pressure.

	Sys. blood pressure			Dia. blood pressure	
	*N*	*β*	95% CI	*β*	95% CI
Unadjusted	101	1.47	0.64, 2.29	0.57	0.14, 1.00
Adjusted with smoking and BMI	101	1.04	0.25, 1.82	0.33	−0.09, 0.74
Adjusted with smoking	101	1.41	0.57, 2.25	0.52	0.08, 0.97
Adjusted with BMI	101	1.05	0.29, 1.81	0.37	−0.03, 0.77
Adjusted with sex	101	1.39	0.58, 2.21	0.54	0.11, 0.96
Adjusted with ethnicity	101	1.52	0.66, 2.38	0.57	0.12, 1.02
Adjusted with education	101	1.37	0.49, 2.24	0.59	0.13, 1.05
Adjusted with all variables^*∗∗*^	101	0.98	0.09, 1.86	0.35	−0.11, 0.81
Men (adjusted with all variables^*∗∗*^)	47	1.07	−0.13, 2.26	0.54	−0.07, 1.14
Women (adjusted with all variables^*∗∗*^)	54	0.68	−0.73, 2.10	0.05	−0.82, 0.72

^*∗∗*^Adjusted for smoking, BMI, sex, ethnicity, and education.

**Table 4:** Linear regression models for lead exposure at various periods of development and mean systolic blood pressure at age 25.

	*N*	*β*	95% CI	*p* value
*Unadjusted*				
Period of exposure				
0–2 years	97	0.007	−0.00, 0.02	0.14
2–4 years	98	0.007	−0.00, 0.01	0.07
4–7 years	92	0.006	0.00, 0.01	0.04
7–12.5 years	89	0.006	0.00, 0.01	0.02
*Adjusted* ^*∗*^				
Period of exposure				
0–2 years	97	0.007	−0.003, 0.018	0.16
2–4 years	98	0.006	−0.001, 0.014	0.11
4–7 years	92	0.005	−0.00, 0.01	0.05
7–12.5 years	89	0.005	0.001, 0.01	0.03

^*∗*^adjusted for ethnicity, sex, and education.

**Table 5:** Linear regression models for concurrent BPb and sVCAM-1 (above) and sICAM-1 (below).

sVCAM-1	*N*	*β*	95% CI
Unadjusted	99	12.92	−1.32, 27.17
Adjusted with ethnicity	99	10.37	−4.44, 25.19
Adjusted with ethnicity and smoking	99	11.15	−4.03, 26.34
Adjusted with ethnicity, smoking, and BMI	99	11.56	−4.21, 27.33
Adjusted with ethnicity, smoking, BMI, and education	99	10.60	−6.22, 27.42
Adjusted with ethnicity, smoking, BMI, education and sex	99	10.53	−6.49, 22.55
Adjusted^1^	47	27.00	9.74, 44.25
Adjusted^2^	52	−16.24	−49.16, 16.67

sICAM-1	*N*	*β*	95% CI

Unadjusted	99	4.76	1.14, 8.38
Adjusted with ethnicity	99	4.22	0.45, 7.99
Adjusted with ethnicity and smoking	99	4.23	0.35, 8.12
Adjusted with ethnicity, smoking, and BMI	99	3.23	−0.62, 7.07
Adjusted with ethnicity, smoking, BMI, and education	99	3.63	−0.46, 7.73
Adjusted with ethnicity, smoking, BMI, education and sex	99	3.63	−0.74, 7.46
Adjusted^1^	47	1.75	−4.44, 7.94
Adjusted^2^	52	5.81	−0.05, 11.68

Adjusted for ethnicity, smoking, BMI, education, and sex. ^1^For men only, adjusted for ethnicity, smoking, BMI, and education. ^2^For women only, adjusted for ethnicity, smoking, BMI, and education. Note regarding the *p* values: sVCAM-1: for men *p* = 0.003, for women *p* = 0.33; sICAM-1: for men *p* = 0.57, for women *p* = 0.052.

## Abbreviations

BPb: Blood lead
sICAM-1: Soluble intercellular adhesion molecule
sVCAM-1: Soluble vascular cell adhesion molecule
BMI: Body mass index
sBP: Systolic blood pressure
dBP: Diastolic blood pressure.

## Supplementary Materials

Supplementary Materials Table: Linear Regression Models for sBP and sVCAM-1.
