# Supplementary material for: Long-Term Effects of Environmental Lead Exposure on Blood Pressure and Plasma Soluble Cell Adhesion Molecules in Young Adults: A Follow-Up Study of a Prospective Cohort in Kosovo
Source: J Environ Public Health. 2018 Jan 8;2018:3180487. doi: 10.1155/2018/3180487 (PMC5817317; doi:10.1155/2018/3180487)
Supplement: Supplementary Materials — Table: Linear Regression Models for sBP and sVCAM-1. [file 3180487.f1.docx]

**Camaj et al**

**Supplemental Table**

Linear Regression Models for sBP and sVCAM-1

**N β 95 % CI**

____________________________________________________________________________________________________________Unadjusted 99 0.00115 -0.00214, 0.00809

Adjusted **^1^** 47 0.00298 -0.00214, 0.00809

Adjusted **^2^** 52 0.00678 -0.01374, 0.00018

A*djusted for ethnicity, smoking, BMI, education, and sex*

**^1^**For men only, *adjusted for ethnicity, smoking, BMI, and education*

**^2^**For women only, *adjusted for ethnicity, smoking, BMI, and education*

Note regarding the p values: sVCAM-1: for men p=0.2468, for women p= 0.0561
